# Hospital-acquired COVID-19 and its effect on length of stay and mortality in orthopedic admissions: A matched cohort study

**DOI:** 10.1016/j.jor.2025.06.031

**Published:** 2025-07-03

**Authors:** Itay Ron, Ammar Muati, David Shaked Zari, Bezalel Peskin, Nabil Ghrayeb, Doron Norman, Jacob Shapira

**Affiliations:** a The Ruth and Bruce Rappaport Faculty of Medicine, Technion - Israel Institute of Technology, Haifa, Israel; b Orthopedic Department, Rambam Medical Center, Haifa, Israel

## Abstract

**Introduction:**

Hospital-acquired COVID-19 poses a significant threat to orthopedic patients, a population already at risk due to immobility, comorbidities, and extended hospital stays. The combined burden of musculoskeletal injury and SARS-CoV-2 infection may prolong recovery, increase complications, and influence survival. This study aimed to evaluate the impact of nosocomial COVID-19 on hospitalization outcomes in orthopedic patients.

**Methods:**

A retrospective cohort study was conducted at a tertiary orthopedic center, analyzing patients hospitalized between 2020 and 2022. COVID-19-positive patients (n = 84) who acquired the infection during admission were matched 1:1 with uninfected controls (n = 84) based on age, gender, and BMI. Data were collected on demographics, comorbidities, hospitalization duration, complications, ICU transfers, and mortality outcomes. Statistical analysis included t-tests, Mann-Whitney U tests, and significance set at p < 0.05.

**Results:**

COVID-19-positive patients experienced significantly longer hospital stays (median 13.9 vs. 4.3 days, p < 0.001) and shorter time to death post-discharge (median 135 vs. 540 days, p = 0.027) compared to controls. Mortality rates were similar between groups (23.8 % vs. 22.6 %, p = 0.86), and ICU admissions occurred only in the COVID-19 group (3.3 %). Baseline characteristics and comorbidity profiles were comparable.

**Conclusion:**

Orthopedic patients who contract COVID-19 during hospitalization face a prolonged hospital course and earlier mortality despite similar overall death rates. These findings highlight the importance of infection prevention strategies, including preoperative screening and deferring elective procedures in infected individuals, to mitigate complications associated with immobility, delayed recovery, and systemic decline.

## 1 Introduction

The COVID-19 pandemic has profoundly impacted global healthcare systems, influencing clinical outcomes across various medical specialties.^1^ Initially recognized as a respiratory illness, COVID-19 has demonstrated far-reaching effects, including cardiovascular, neurological, and musculoskeletal complications.^2^ The interplay between SARS-CoV-2 infection and pre-existing conditions among hospitalized patients remains poorly understood. Emerging evidence highlights that COVID-19 exacerbates systemic inflammation and coagulation abnormalities, which may increase the risk of complications in hospitalized patients.^3^

This is particularly concerning for orthopedic patients, a population uniquely vulnerable due to immobility, comorbidities, and the risk of nosocomial infections during extended hospital stays.^4^ Specifically, immobilized patients are at increased risk for thromboembolic events, such as deep vein thrombosis (DVT) and pulmonary embolism, risks that are further exacerbated by the hypercoagulable state associated with COVID-19.^5^ Moreover, prolonged hospitalization and immobility contribute to muscle deconditioning, delayed wound healing, and a heightened susceptibility to infections.^6^, ^7^, ^8^

Emerging evidence suggests that these patients may experience prolonged recovery times, higher rates of surgical site infections, and an increased risk of ICU admission compared to their non-COVID-19 counterparts.^9^^,^^10^ This effect may be due to the necessity of managing the acute effects of the virus while simultaneously addressing their underlying musculoskeletal conditions.^11^ Nevertheless, data specifically examining the impact of nosocomial COVID-19 infections on the clinical course and outcomes of orthopedic patients remains limited.

This study aimed to evaluate the influence of hospital-acquired COVID-19 on the hospitalization course and outcomes of orthopedic patients. By comparing the clinical trajectories of patients who contracted COVID-19 during their orthopedic admission with those who remained uninfected, this study aimed to identify potential differences in hospitalization duration, complication rates, recovery processes, and critical care utilization.

## 2 Methods

This retrospective cohort study included data from an orthopedic department at a tertiary trauma center. The hospital database was used to identify the relevant patients; a search was made through the MDClone ADAMS system, integrated with our electronic medical records, a self-service data analytics environment enabling the generation of complex search queries and easy access to all retrospective data within the hospital. Eligible patients were orthopedic patients older than 18 who were hospitalized at the orthopedic department between 2020 and 2022. Exclusion criteria were pregnant women and patients with incomplete medical records. Data was analyzed to find patients who acquired COVID-19 during their hospitalization, and a matching process was made to minimize cofounding factors. Patients infected with COVID-19 during their hospitalization were matched 1:1 to patients without COVID-19; matching was based on age, gender, and BMI to ensure comparable baseline characteristics between the groups. The evaluated variables were demographics (i.e., age, gender), comorbidities, hospitalization duration, complications during hospitalization, transfers to intensive care units (ICU), and time to death post-hospitalization. All patients had a minimum follow-up time of 2 years.

Descriptive statistics for mean, standard deviation, median, percentages, and ranges were performed for all parameters. The normal distribution of continuous parameters was tested using the Kolmogorov-Smirnov test. As a result of this test, we used *t*-test or Mann-Whitney U tests to determine the differences between the two groups (COVID vs. control). As the cohort of the control group was huge (n = 10147) and the COVID-19 group was 84 patients, we used the Matching technique (match by age) to divide the total control group into a 1:1 ratio (study: control group), which included 84 patients in the control and study groups. P < 0.05 was considered significant. SPSS version 28 was used for all statistical analyses.

IRB approval was obtained from our hospital's ethical committee, with a waiver for informed consent due to the study's retrospective nature. Data were handled in compliance with institutional and international guidelines for medical research, ensuring anonymity and data security.

## 3 Results

**Fig. 1:**
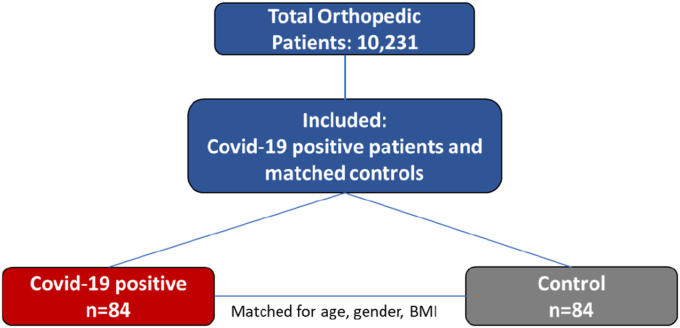
Flowchart of patient selection and 1:1 matching by age, gender, and BMI

The initial search yielded 10,231 patients admitted to the orthopedic department between 2020 and 2022. Of them, 84 patients were positive for COVID-19 during their hospitalization. These patients were matched 1:1 with 84 control patients based on age, gender, and BMI (Fig. 1). A total of 168 patients were included in the analysis. The mean age was similar between groups (73.4 ± 16.1 vs. 70.3 ± 15.7 years, p = 0.21), as were gender distributions (50 % male in the COVID-19 group vs. 38 % in the control group, p = 0.12) and BMI (27.4 ± 5.33 vs. 26.6 ± 4.3, p = 0.55). Mortality rates were comparable (23.8 % vs. 22.6 %, p = 0.86), with no significant differences in smoking status (10.7 % vs. 14.3 %, p = 0.48) or Charlson Comorbidity Score (3.36 ± 2.58 vs. 3.33 ± 2.45, p = 0.94). Regarding transfers to intensive care units (ICU), only three patients were transferred, and all of them were from the COVID-19 group (3.3 % vs. 0 %, p = 0.25) (Table 1).

**Table 1:** Baseline characteristics and outcomes of COVID-19 positive and control groups.

	Control 1; n = 84	Covid; n = 84	p
Age	70.3 ± 15.7	73.4 ± 16.1	P = 0.21
Gender			P = 0.12
Male	32 (38 %)	42 (50 %)	
Female	52 (62 %)	42 (50 %)	
BMI; 13–50	26.6 ± 4.3	27.4 ± 5.33	P = 0.55
Deceased date	19 (22.6 %)	20 (23.8 %)	P = 0.86
Smoking	12 (14.3 %)	9 (10.7 %)	P = 0.48
Charlson Comorbidity Score Post-Hospitalization	3.33 ± 2.45	3.36 ± 2.58	P = 0.94
Transfers to ICU-department	0	3 (3.3 %)	P = 0.25

This table presents the demographic and clinical characteristics of 168 patients, divided into 84 COVID-19-positive patients and 84 matched controls. Variables include age, gender distribution, body mass index (BMI), mortality rates, smoking status, Charlson Comorbidity Score post-hospitalization, and ICU transfer rates. No significant differences were observed in age (p = 0.21), gender distribution (p = 0.12), BMI (p = 0.55), mortality rates (p = 0.86), smoking status (p = 0.48), or Charlson Comorbidity Score post-hospitalization (p = 0.94). ICU transfers occurred exclusively in the COVID-19-positive group (3.3 %, p = 0.25). These findings highlight the comparable baseline characteristics between groups, with notable differences in ICU transfer rates observed.

The prevalence of pre-existing comorbidities was assessed for both groups. Rates of hypertension were similar between COVID-19-positive patients and controls (38.1 % vs. 40.5 %; p = 0.75), as were rates of cerebrovascular disease (CVA, 4.8 % vs. 2.4 %; p = 0.68), chronic obstructive pulmonary disease (COPD, 4.8 % vs. 6.0 %; p = 1.00), and myocardial infarction (MI, 9.5 % vs. 6.0 %; p = 0.38) (Table 2).

**Table 2:** Baseline prevalence of comorbidities in COVID-19 positive and control groups.

Background Disease	Control 1; n = 84	Covid; n = 84	p
Hypertension	34 (40.5 %)	32 (38.1 %)	P = 0.75
CVA	2 (2.4 %)	4 (4.8 %)	P = 0.68
COPD	5 (6.0 %)	4 (4.8 %)	P = 1.00
MI	5 (6.0 %)	8 (9.5 %)	P = 0.38

This table shows the prevalence of key comorbid conditions (hypertension, cerebrovascular disease, chronic obstructive pulmonary disease, and myocardial infarction) in both groups. No statistically significant differences were observed between the groups.

**Fig. 2:**
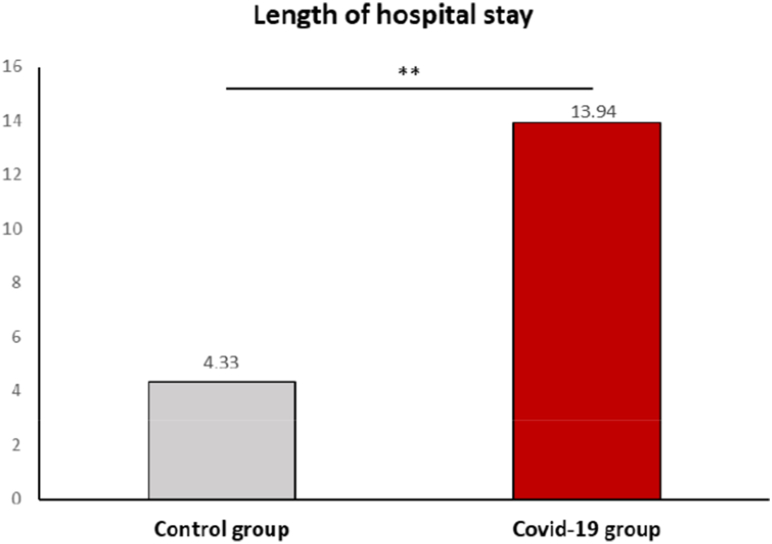
Median Hospital Length of Stay in COVID-19 Positive and Control Groups. This bar plot compares the median hospital length of stay between COVID-19-positive patients and the control group. The median stay for COVID-19 patients was 13.94 days, significantly longer than the 4.33 days observed in the control group (p < 0.001).

The length of hospital stay (LOS) was significantly longer for COVID-19-positive patients compared to the control group (Fig. 2). COVID-19 patients had a median hospitalization duration of 13.94 days (IQR: 7.3–20.9), which was more than three times the duration observed in the control group (4.33 days, IQR: 1.2–8.7; p < 0.001).

**Fig. 3:**
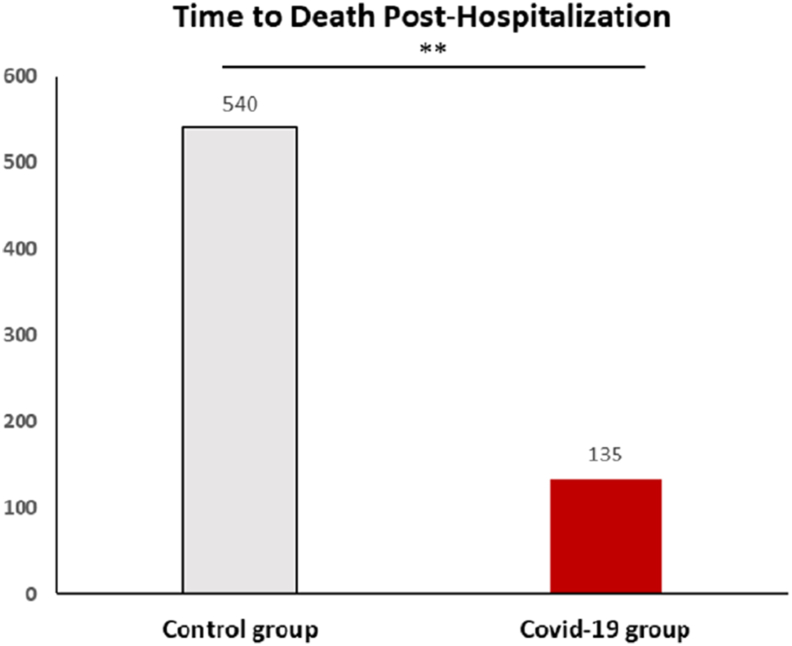
Median Time to Death Post-Hospitalization for COVID-19 Positive and Control Groups This bar plot compares the median time to death post-hospitalization between COVID-19-positive patients and the control group. The median time to death was significantly shorter in the COVID- 19-positive group (135 days, IQR: 28–552) compared to the control group (540 days, IQR: 126–1065; p = 0.027), reflecting a more acute disease progression in COVID-19-positive patients.

There was no difference between the groups regarding mortality rate (22.6 % vs 23.8 %; p = 0.86). Among deceased patients, the time to death post-hospitalization was significantly shorter for COVID-19-positive patients compared to the control group (Fig. 3). COVID-19 patients had a median time to death of 135 days (IQR: 28–552), while control patients had a significantly longer median time of 540 days (IQR: 126–1065; p = 0.027).

## 4 Discussion

This study aimed to assess the impact of hospital-acquired COVID-19 on orthopedic patients, analyzing clinical outcomes such as hospitalization duration, mortality, and ICU admissions. It found that orthopedic patients who acquired COVID-19 during admission were hospitalized three times longer than non-infected patients. Even though both groups had the same mortality rate, COVID-19 patients had a significantly shorter time to death post-hospitalization.

One of the objectives of this study was to evaluate the mortality rate of orthopedic patients infected with COVID-10 during their hospitalization. A study by Kinsely et al. examined 468 patients who underwent urgent or emergent surgery; approximately 8 % of the patients were diagnosed with COVID-19 peri-operatively.^12^ In their study, the perioperative mortality rate was 16.7 % in those with COVID-19 compared to 1.4 % in COVID-19 negative subjects. A multi-center cohort study by Nepogodiev et al. analyzed 1128 patients who had surgery during the COVID pandemic; their results suggested that postoperative pulmonary complications occur in half of patients with perioperative SARS-CoV-2 infection and are associated with high mortality.^13^ Another systematic review concluded an unexpectedly high postoperative mortality rate in SARS-CoV-2-infected patients of 20 % in the global literature.^14^ Opposing current literature, in this matched cohort, overall mortality rates were comparable between COVID-19 and non-COVID-19 groups; however, the median time to death was significantly shorter in the COVID-19 group (135 days vs. 540 days), suggesting a more rapid clinical decline following infection. These findings underscore the importance of early identification of hospital-acquired COVID-19 and highlight the need for vigilant monitoring and timely, tailored interventions to mitigate long-term adverse outcomes in this high-risk patient population.

This study evaluated the length of hospital stay to measure complications, recovery, and overall healing in patients admitted to the orthopedic department. A common approach is to reduce hospitalization LOS to reduce complications and morbidities related to hospital stay.^15^ Studies suggest that deconditioning makes older orthopedic patients with multiple comorbidities more prone to long stays.^16^ A recent systematic review evaluated the length of hospital stay of patients suffering from COVID-19, which showed that COVID-19 patients were hospitalized for more than 10 days, and the duration of hospitalization was dependent on factors such as age.^17^ This study found similar results as COVID-19 patients had a median hospitalization duration of almost 14 days, more than three times the duration observed in the control group. These findings align with existing literature emphasizing the importance of reducing hospital length of stay, as prolonged hospitalization is associated with increased risk of immobility-related complications, hospital-acquired infections, and delayed recovery. This study underscores the need for targeted clinical strategies for orthopedic patients with concurrent COVID-19 infection—a population facing a dual challenge of requiring early mobilization and rehabilitation, while being prone to extended hospital stays and higher complication rates. Optimizing perioperative care, discharge planning, and early rehabilitation protocols for this vulnerable subgroup may improve outcomes and reduce healthcare burden.

This study has several limitations. First, its retrospective design inherently introduces a risk of selection bias and susceptibility to unmeasured confounding factors, as data were collected from existing records rather than through prospective enrollment. Second, although the matching process was used to reduce confounding, it may have introduced overmatching and limited the ability to assess the effect of variables used for matching. Moreover, matching may have excluded patients without suitable counterparts, potentially reducing the sample size and statistical power. Third, the findings may not be generalizable to the broader population, as the matched cohort represents a highly selected group and may not reflect the diversity of clinical presentations or management strategies in real-world settings. Finally, due to the nature of the dataset, certain clinical parameters such as functional outcomes, rehabilitation adherence, or socioeconomic variables were not available for analysis and may have influenced the results.

In conclusion, orthopedic patients who acquire COVID-19 during hospitalization face prolonged stays and a shorter time to death, likely due to reduced resilience to both surgical stress and viral illness. These findings highlight the need for early detection and preventive strategies, including routine preoperative screening and postponement of elective procedures, to reduce complications from delayed mobilization, impaired healing, and hospital-acquired morbidity.

## CRediT authorship contribution statement

**Itay Ron:** Writing. **Ammar Muati:** data curing. **David Shaked Zari:** Writing – review & editing. **Bezalel Peskin:** Conceptualization, Methodology, Formal analysis. **Nabil Ghrayeb:** Conceptualization, Methodology, Formal analysis. **Doron Norman:** Conceptualization, Methodology, Formal analysis. **Jacob Shapira:** Writing – review & editing, Conceptualization, The authors received no financial support for the research, authorship, or publication of this article.

## Ethics approval and consent to participate

All data collection and reporting received institutional review board approval.

Due to its retrospective and non-interventional nature, written informed consent was waived.

## Funding

The authors received no financial support for the research, authorship, or publication of this article.

## Conflict of interest

All authors have no competing interests.
